# Ultra-high sensitivity gas sensors employing Bloch-like surface waves in a metal-dielectric one-dimensional photonic crystal

**DOI:** 10.1038/s41598-026-38689-z

**Published:** 2026-02-09

**Authors:** Michal Gryga, Jakub Chylek, Dalibor Ciprian, Petr Hlubina

**Affiliations:** Department of Physics, Technical University Ostrava, 17. listopadu 2172/15, 708 00 Ostrava-Poruba, Czech Republic

**Keywords:** Metal-dielectric one-dimensional photonic crystal, Bloch surface waves, Reflectance, Gas sensor, Ultra-high sensitivity, Materials science, Optics and photonics, Physics

## Abstract

Bloch surface waves (BSWs), generated at the interface of a truncated one-dimensional photonic crystal (1DPhC) and the adjacent medium (analyte), are accompanied by narrow resonance dips that are very advantageous compared to too wide resonance dips associated with the surface plasmon resonance (SPR) phenomenon. Consequently, BSW-based sensors have been thoroughly studied and applied in the field of optical sensors, but their sensitivity to gaseous analytes does not outperform the sensitivity of the SPR-based sensors. One of the possible solutions to enhance the sensitivity represents a metal-dielectric 1DPhC. We report on a sensing concept for gaseous analytes based on the wavelength interrogation and resonances supported by a metal-dielectric 1DPhC in the Kretschmann configuration. For a metal-dielectric 1DPhC comprising bilayers of TiO$_2$/Au with a termination layer of TiO$_2$, we show that the Bloch-like SW-based resonances are resolved for both TE and TM waves. For the TE wave and the refractive index (RI) in a range of 1–1.0015, a sensitivity of 10,900 nm/RIU, a figure of merit (FOM) of 474 RIU$^{-1}$, and a limit of detection (LOD) of 9.3 $\times$ 10$^{-6}$ RIU were reached. The analysis extended to the 1DPhC with the modified thicknesses of TiO$_2$ layers and gas, whose RI changes in a range of 1.0002–1.0022, leads to the sensitivity and FOM in a range of 10,680–28,000 nm/RIU and 434–1217 RIU$^{-1}$, respectively, and to a very low LOD of 3.6 $\times$ 10$^{-6}$ RIU for the TM wave. This research is the demonstration of exceptional properties of the Bloch-like SW-based sensors employing metal-dielectric 1DPhCs that can be used in a simple sensing of a wide range of gaseous analytes.

## Introduction

Complex dielectric structures^1^ with periodically varying dielectric constant such as one-dimensional photonic crystals (1DPhCs)^2^ are characterized by a photonic band gap (PBG), and have been exploited as optoelectronic devices, including omnidirectional reflectors^3^, polarization selectors^4^, narrow band optical filters^5^, light emitters^6^, tunable lasers^7^ and optical sensors^8^. If a periodic, semi-infinite structure is composed of bilayers of dielectric media of high and low refractive indices (RIs), and a termination layer as well, a 1DPhC is formed. In such a system, the optical waves exist that are gradually enhanced with the maximum amplitude near the interface with the outer medium (analyte). Such waves are referred to as Bloch surface waves (BSWs), and they enable high optical field confinement and thus narrow resonance dips within the PBG of the reflection spectrum, and are preferred in the realization of various types of optical sensors^9–22^ and other applications^22^. Narrow BSW-based resonances are advantageous compared to wide resonances accompanied by the surface plasmon resonance (SPR) phenomenon^23–27^. Contrary to SPR-based sensors, BSWs can be excited by both TE and TM waves^28,29^ at any wavelength, including also near- or mid-infrared regions^20,30^, and this working principle can be attained by suitably changing the geometry and materials of the 1DPhC.

BSW-based sensors, that exhibit sharp resonances, are also advantageous because they do not rely on the use of metals. Moreover, these sensors are mechanically and chemically robust, offering the possibility of operation in aggressive environments. However, the BSW-based sensors are outperformed by SPR-based ones in sensitivity. To attain the comparable sensitivity, the 1DPhCs with high field confinement and enhancement, and long penetration depth in the analyte need to be designed, especially when they are used in gaseous analytes sensing.

Because both SPR- and BSW-based sensors cannot be free-space excited, suitable alternatives have been proposed and realized, such as ones using confined electromagnetic modes called Tamm plasmons (TPs)^31–33^ or cavity modes (CMs)^34–39^. Tamm plasmon is generated when an all-dielectric 1DPhC is extended by a thin metal layer, and the optical wave is gradually enhanced in the 1DPhC, but with the maximum amplitude near the interface of the 1DPhC and metal layer. TPs and CMs can be excited at any angle of incidence by both TE and TM waves and these advantageous concepts have led to a number of applications in the field of optical sensors^40–49^, including gas sensors^34–39,46–49^. The gas sensors have been operated in the angular^46^ or spectral domain^34–38,47–49^ and can reach sensitivities of 2 $\times 10^{5}$ deg/RIU^46^, 700 nm/RIU^34^, 1200 nm/RIU^35,36^, 52,300 nm/RIU^38^, and 190,000 nm/RIU^47^, respectively. However, the CM-based sensors^38^ have some experimental limitations^39^. Similarly, some limitations of TP-based sensors are related to the fact that the confined optical field is located within the structure near the metal-dielectric interface, and the analyte needs to reach the detection volume^42^. One of possible solutions to fulfill this requirement is using porous 1DPhCs^42–44,47,48^. The high specific surface of such materials is very advantageous in binding the molecules and thus in gas sensing applications^47^, but the response time is awaited to be prolonged due to this sensing concept.

When the metallic layers instead of low-RI dielectric layers are incorporated into a periodic structure of a 1DPhC, a metal-dielectric 1DPhC characterized by an extremely high refractive index contrast within the unit cell is obtained^50–52^. Real 1DPhC is in fact photonic crystal slab—the structure truncated on both sides, and such a system is referred to as a truncated 1DPhC. When the truncated 1DPhC contains only a few bilayers, the concept of the BSW, originally developed for a semi-infinite number of bilayers, can not be used in a straightforward manner. Moreover, when a metal-dielectric 1DPhC is considered, the problem is more complicated. Nevertheless, there can exist some SWs that share with BSWs an important feature for sensing - they are localized near the interface with an analyte. We call them the Bloch-like SWs. These SWs exhibit a lot of interesting properties, however, the sensitivity to the RI of liquid analytes is not too high^52^. In addition, compared to the all-dielectric 1DPhC, the metal layers in a 1DPhC (a metal-dielectric 1DPhC) lead to the extremely high RI contrast and to further field enhancement on the mentioned surface.

In this paper, the sensing concept based on the Bloch-like SW excitation is extended to gaseous analytes and the corresponding resonances supported by a metal-dielectric 1DPhC in the Kretschmann configuration are employed in sensors with an ultra-high sensitivity. We use the wavelength interrogation and show for a metal-dielectric 1DPhC, comprising different numbers of bilayers of TiO$_2$/Au with a termination layer of TiO$_2$, that the Bloch-like SW resonances are resolved for both TE and TM waves when the RI is in a range of 1–1.0015. When two bilayers of TiO$_2$/Au are used, the sensitivity, figure of merit (FOM), and limit of detection (LOD) for the TE wave reach 10,900 nm/RIU, 474 RIU$^{-1}$, and 9.3 $\times$ 10$^{-6}$ RIU, respectively. The analysis extended to the 1DPhC with the modified thicknesses of TiO$_2$ and gas of RI in a range of 1.0002–1.0022 revealed that the sensitivity and FOM in a range of 10,680–28,000 nm/RIU and 434–1217 RIU$^{-1}$, respectively, and a very low LOD of 3.6 $\times$ 10$^{-6}$ RIU were reached for the TM wave. This sensing concept is demonstrative for optical sensors with an ultra-high sensitivity to gaseous analytes. It is advantageous because porous layers are not employed so that the response time is awaited to be as short as possible. Finally, sensors employing a metal-dielectric 1DPhC supporting Bloch-like SWs can be used in a simple sensing of a wide range of gaseous analytes.

## Theoretical model and basic equations

**Fig. 1:**
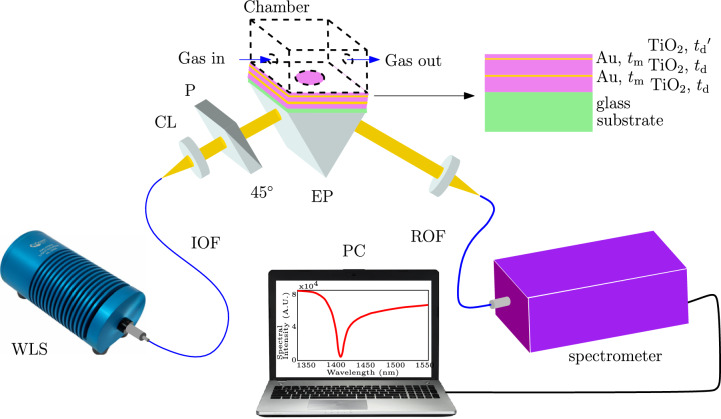
Schematic drawing of the experimental setup employing a metal-dielectric 1DPhC; white light source (WLS), input optical fiber (IOF), collimating lens (CL), polarizer (P), equilateral prism (EP), microscope objective (MO), read optical fiber (ROF) and personal computer (PC).

The metal-dielectric 1DPhC under study, whose optical response to a gaseous analyte is analyzed by employing the wavelength interrogation in the Kretschmann configuration shown in Fig. 1, is drawn schematically in the same figure. It consists of a glass substrate, titanium dioxide layers and gold layers.

Both a prism and substrate are supposed to be made from BK7 glass and its refractive index as a function of wavelength can be described by a three-term Sellmeier formula^53^:

1 $$\begin{aligned} n^2(\lambda ) = 1 + \sum _{i=1}^{3} \frac{A_i\lambda ^2}{\lambda ^2-B_i}, \end{aligned}$$

where the wavelength $\lambda$ is in $\mu$m and the values of Sellmeier coefficients at the room temperature are as follows: $A_1$ = 1.03961212, $A_2$ = 2.31792344$\times 10^{-1}$, $A_3$ = 1.01046945, $B_1$ = 6.00069867$\times 10^{-3} \mu$m$^2$, $B_2$ = 2.00179144$\times 10^{-2} \mu$m$^2$ and $B_3$ = 1.03560653$\times 10^{2}$ $\mu$m$^2$. Similarly, the dispersion relation for the TiO$_2$ layer is given by^29^

2 $$\begin{aligned} n^2(\lambda )=A+\frac{B\lambda ^2}{\lambda ^2-C^2}, \end{aligned}$$

where constants *A*, *B* and *C* are with values $A=2.7655$, $B=2.2$, $C=0.26524$ $\mu$m.

The material dispersion of the Au layer can be described by the complex dielectric function given by the Drude–Lorentz model^54^:

3 $$\begin{aligned} \varepsilon _{\textrm{Au}}(\lambda ) = \varepsilon _{\infty } - \frac{1}{\lambda _p^2 (1/\lambda ^2 + i/\gamma _p \lambda )} - \sum _{j=1}^2\frac{A_j}{\lambda _j^2(1/\lambda ^2 - 1/\lambda _j^2) + i\lambda _j^2/\gamma _j\lambda }. \end{aligned}$$

with parameters presented in previous papers^55,56^. This model takes into account the interband transitions and is also applicable in near-IR range^57^.

Light propagation through a metal-dielectric 1DPhC in the Kretschmann configuration can be analyzed by the transfer matrix method (TMM)^58,59^. The method enables to express the optical response, such as the spectral reflectances $R_{s,p}(\lambda )$ for both *s*-polarized (transverse electric, TE) and *p*-polarized (transverse magnetic, TM) waves, using the fact that electric or magnetic fields in one position can be related to those in other positions through a transfer matrix. If *N* layers are considered, including both metal and dielectric media, the transmission matrices across different interfaces and propagation matrices in different layers enable to express the total transfer matrix $\textbf{M}(\lambda )$ at the wavelength $\lambda$ as:

4 $$\begin{aligned} \textbf{M}(\lambda )= \left[ \begin{array}{cc} M_{11}(\lambda ) & M_{12}(\lambda ) \\ M_{21}(\lambda ) & M_{22}(\lambda ) \end{array} \right] = \left[ \prod \limits _{j=1}^{N} \boldsymbol{\mathrm B}_{j-1,j}(\lambda ) \boldsymbol{\mathrm P}_j (\lambda ) \right] \cdot \boldsymbol{\mathrm B}_{N,N+1}(\lambda ), \end{aligned}$$

where indices 0 and $N+1$ denote the first and last semi-infinite medium, respectively, and $\textbf{B}_{j,j+1}(\lambda )$ are the boundary matrices for *s*- and *p*-polarized waves that satisfy the relations^60^

5 $$\begin{aligned} \textbf{B}_{j,j+1}(\lambda )=\frac{1}{2}\left( \begin{array}{cc} 1+\tilde{\eta }_{s,p}(\lambda ) & 1-\tilde{\eta }_{s,p}(\lambda )\\ 1-\tilde{\eta }_{s,p}(\lambda ) & 1+\tilde{\eta }_{s,p}(\lambda ) \\ \end{array} \right) , \end{aligned}$$

where the complex parameters $\tilde{\eta }_s(\lambda )$ and $\tilde{\eta }_p(\lambda )$ are given by

6 $$\begin{aligned} \tilde{\eta }_s(\lambda )=\frac{k_{zj+1}}{k_{zj}},~~~~~~~~~\tilde{\eta }_p(\lambda )=\frac{\tilde{n}_{j}^2(\lambda )k_{zj+1}}{\tilde{n}_{j+1}^2(\lambda )k_{zj}}, \end{aligned}$$

where $\tilde{n}_{j}(\lambda )$ and $\tilde{n}_{j+1}(\lambda )$ are the complex refractive indices of the media and

7 $$\begin{aligned} k_{zj}(\lambda )=\left[ \left( \tilde{n}_{j}(\lambda )\frac{\omega }{c}\right) ^{2}-(n_{0}(\lambda )\frac{\omega }{c}\sin {\theta })^{2}\right] ^{1/2}. \end{aligned}$$

Similarly, the propagation matrices are given by

8 $$\begin{aligned} \textbf{P}_{j}(\lambda )=\left( \begin{array}{cc} e^{\text {i}k_{zj}(\lambda )t_{j}}& 0\\ 0 & e^{-\text {i}k_{zj}(\lambda )t_{j}} \\ \end{array} \right) , \end{aligned}$$

where $t_{j}$ is the thickness of *j*-th layer.

The complex reflection coefficient $r(\lambda )$ is related to the total transfer matrix elements by

9 $$\begin{aligned} r(\lambda ) = \frac{M_{21}(\lambda )}{M_{11}(\lambda )}, \end{aligned}$$

and the spectral reflectances $R_{s,p}(\lambda )$ for both *s* and *p* polarizations can be calculated as

10 $$\begin{aligned} R_{s,p}(\lambda )=\left| r_{s,p}(\lambda )\right| ^2. \end{aligned}$$

In order to explain the theoretical optical reflection/transmission spectra of thin film structures containing periodic layers, the PhC concepts are usually used. The so-called band diagram plays the key role here, as it can give the information about the nature of various resonances that can occur in modeled/measured spectra. Here, it is important to note that the concept of BWs or BSWs presumes an infinite or semi-infinite structure. Infinite number of some periodic pattern, for example bilayers in a 1DPhC, leads to the existence of allowed and forbidden bands (referred to as band gaps). When a one half of the infinite structure is replaced by a homogeneous medium, some additional lines can occur in the band gaps and these correspond to BSWs. To confirm that the resonance observed in reflection/transmission spectrum can be attributed to some BSWs, one can simply check, if the excitation condition leads to the point somewhere in the band gap. In contrast, the prepared 1DPhCs always contain finite number of bilayers. Thus care has to be taken when the explanation based on BWs/BSWs is used. The classification based on band diagram can be supported by computation of the field profile in the analyzed structure with finite extent.

Considering the 1DPhC system containing only loss-less media, the band diagram can be easily computed as the criteria for the band edges are well known^61^. On the other hand, in the case of a metal-dielectric 1DPhC (generally, the structure contains lossy layers), the problem of band diagram computing has not been solved yet in full extent. The problem is that the Bloch wavenumber^61^ *K* in system is intrinsically complex, contrary to the loss-less case, where *K* is always either real (allowed band) or pure imaginary (band gap). In such a case, the band edges are well defined. As to the metal-dielectric 1DPhCs, no reasonable definition of band edges exists (as to the authors’ knowledge). To deal with such a situation, some heuristic approach can be suggested. It is based on the assumption that the computed reflection spectrum tends to a “stable” pattern, when the number of bilayers is increased. As it closely corresponds to the band diagram (not accessible here), one can try to locate the band gaps from reflection/transmission spectrum shape obtained for model system containing satisfactory number of bilayers. Then some criteria can be suggested to find the band “edges” and to apply the usual photonic crystal terms to explain the nature of the analyzed spectra. The existence of Bloch-like SWs can be checked out only by the field profile computation, where the reflection/transmission resonances are obtained. This approach is used in following text.

## Fabrication of metal-dielectric 1DPhCs

To manufacture metal-dielectric 1DPhCs, different techniques can be used. Glass substrates can be coated homogeneously by a deposition technique based on the radio frequency (RF) magnetron sputtering^62^ with TiO$_2$ and Au. TiO$_2$ and Au targets with purity as high as possible need to be employed, when the working gas of argon is used under the suitable deposition pressure and forward RF power. Moreover, TiO$_2$ layers can be deposited in the mixture of Ar and O$_2$ under the suitable total deposition pressure and partial oxygen pressure, and forward RF power. To control the fabrication process, methods of spectral ellipsometry^21^ and atomic force microscopy^56^ can be utilized to determine the thicknesses of individual layers and their surface roughness. Moreover, alternatives such as e-beam deposition technique^63^ can be applied.

## Theoretical results and discussion

First, we analyzed the spectral response of the metal-dielectric 1DPhC for the angle of incidence in a range of $35^\circ$–$85^\circ$ when the surrounding medium (analyte) is with the RI $n=1$ and when two or three bilayers of TiO$_2$/Au with a termination layer of TiO$_2$ are considered. Taking into account the dispersion of the prism and materials of the 1DPhC specified above, and assuming that the extinction coefficient for TiO$_2$ is with a value of 1.6$\times 10^{-3}$, as specified in previous papers^10,29^, the most pronounced dips in the reflectance spectrum were obtained for Au thickness $t_\textrm{m}=14$ nm, TiO$_2$ thickness $t_\textrm{d}=380$ nm and the termination layer thickness $t_\textrm{d}{'}=180$ nm.

**Fig. 2:**
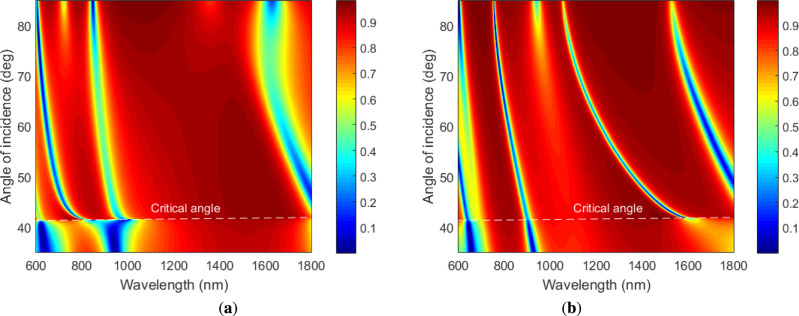
Calculated contour plots of the reflectance as a function of the angle of incidence and wavelength for TM wave (**a**) and TE wave (**b**) when two bilayers of TiO$_2$/Au are considered. The analyte RI $n=1$.

**Fig. 3:**
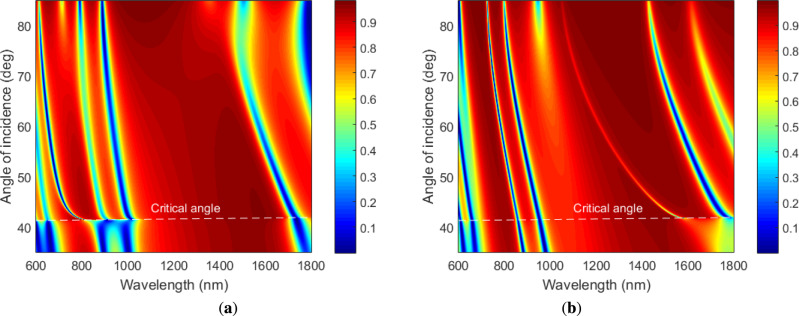
Calculated contour plots of the reflectance as a function of the angle of incidence and wavelength for TM wave (**a**) and TE wave (**b**) when three bilayers of TiO$_2$/Au are considered. The analyte RI $n=1$.

In order to have extensive insights on the modes propagating in the metal-dielectric 1DPhC, we calculated contour plots of the reflectance as a function of the angle of incidence and wavelength for both TM and TE waves, which correspond to the dispersion of the different modes for both polarization states. From Fig. 2a,b, corresponding to two bilayers of TiO$_2$/Au, it is clear that different number of modes are present, and some of them are at angles smaller than the critical angle, and they correspond to guided modes. Moreover, two modes up to the critical angle are present and Fig. 2a,b, and indicate that the TE mode shows the smaller width than the TM mode. When three bilayers of TiO$_2$/Au are considered, contour plots of the reflectance shown in Fig. 3a,b indicate that greater number of modes are present and once again two modes are up to the critical angle. The TE mode has the smaller width than the TM mode.

**Fig. 4:**
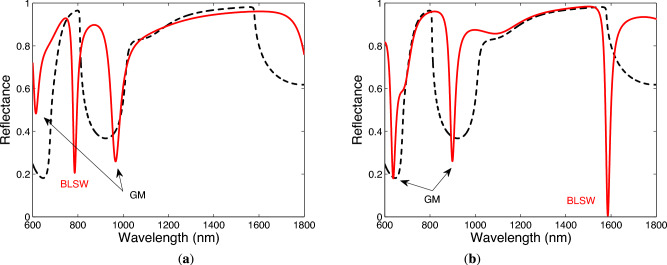
Theoretical spectral reflectances for TM (**a**) and TE (**b**) waves when two bilayers of TiO$_2$/Au are considered (BLSW—Bloch-like SW, GM—guided mode). The angle of incidence $\theta =41.9^\circ$ and the analyte RI $n=1$.

Based on the calculated contour plots of the reflectance as a function of the angle of incidence and wavelength for both TM and TE waves, we choose the angle of incidence $\theta =41.9^\circ$, and Fig. 4a,b show the corresponding theoretical spectral reflectances, when two bilayers of TiO$_2$/Au are considered. Figure 4a shows several dips resolved for TM wave in the considered wavelength range, with the most narrow dip at a wavelength of 785.7 nm. Similarly, Fig. 4b shows dips resolved for TE wave, and the most narrow dip with the maximum depth is at a wavelength of 1585.9 nm. In addition, the spectral reflectances were computed for hundred bilayers of TiO$_2$/Au and are shown in Fig. 4a,b by the dashed lines. Both the dips are within the band gaps of the 1DPhC whose “edges” were estimated under the condition that the reflectance has a value of 0.75, and these resonances can be associated with Bloch-like SWs.

**Fig. 5:**
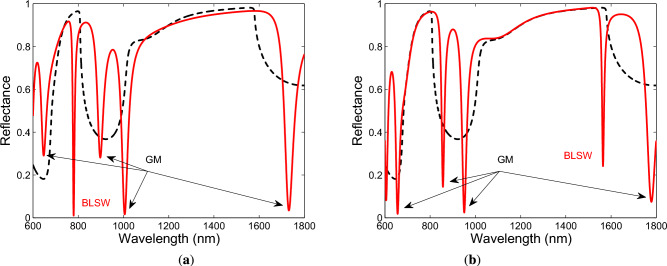
Theoretical spectral reflectances for TM (**a**) and TE (**b**) waves when three bilayers of TiO$_2$/Au are considered (BLSW—Bloch-like SW, GM—guided mode). The angle of incidence $\theta =41.9^\circ$ and the analyte RI $n=1$.

When three bilayers of TiO$_2$/Au are considered, the theoretical spectral reflectances for both TM and TE waves are shown in Fig. 5a,b, and the reflectance spectra exhibit more dips. In addition, the most narrow dips are once again associated with the Bloch-like SWs, for TM wave resolved at a wavelength of 779.7 nm and for TE wave at a wavelength of 1564.4 nm. When comparing the results with those for two bilayers of TiO$_2$/Au, both dips are shifted to the shorter wavelengths and thus are localized more within the band gaps, and are also much narrower, but the dip for TE wave is with the smaller depth.

**Fig. 6:**
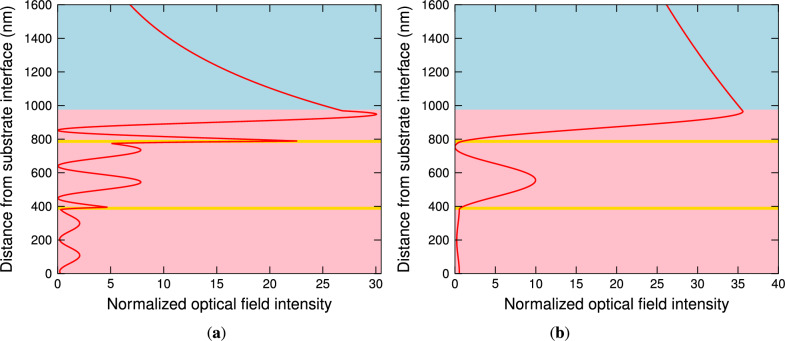
The normalized optical field intensity distribution for TM wave at a wavelength of 785.7 nm (**a**) and for TE wave at a wavelength of 1585.9 nm (**b**) when two bilayers of TiO$_2$/Au are considered. The angle of incidence $\theta =41.9^\circ$ and the analyte RI $n=1$.

To support the statement that both the resonances are related to the Bloch-like SWs, the optical field intensities normalized to the incident field intensities are computed. In Fig. 6a, the normalized optical field intensity $\left| H_x\right| ^2/\left| H_{x0}\right| ^2$ in the structure is shown for TM wave at a wavelength of 785.7 nm, which is related to the Bloch-like SW resonance because the envelope of the field is exponentially increasing in the 1DPhC and an exponential tail in the analyte is also apparent. The computation was performed using the TMM^64^ and $H_{x0}$ is *x* component of the incident magnetic field, which is perpendicular to the plane of incidence. Similarly, the normalized optical field intensity $\left| E_x\right| ^2/\left| E_{x0}\right| ^2$ for TE wave at a wavelength of 1585.9 nm is shown in Fig. 6b, and once again it can be considered as a very good manifestation of the Bloch-like SW excitation because the envelope of the field, which is of a higher amplitude, is exponentially increasing in the 1DPhC and exponentially decreasing in the analyte. These figures clearly demonstrate that the enhanced optical field, characterized by more than a twenty nine-fold enhancement of the optical intensity with respect to the incident beam, is confined to the surface of the 1DPhC.

**Fig. 7:**
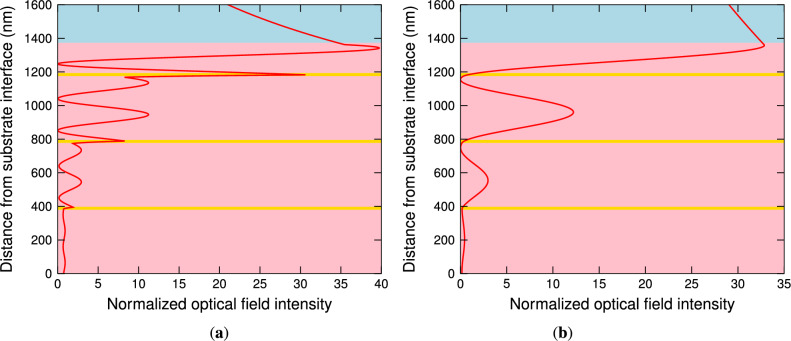
The normalized optical field intensity distribution for TM wave at a wavelength of 779.7 nm (**a**) and for TE wave at a wavelength of 1564.4 nm (**b**) when three bilayers of TiO$_2$/Au are considered. The angle of incidence $\theta =41.9^\circ$ and the analyte RI $n=1$.

The narrower dips are due to the stronger field in the analyte, as demonstrated in Fig. 7a,b. In Fig. 7a, the normalized optical field intensity $\left| H_x\right| ^2/\left| H_{x0}\right| ^2$ in the structure is shown at a wavelength of 779.7 nm, and similarly, the normalized optical field intensity $\left| E_x\right| ^2/\left| E_{x0}\right| ^2$ at a wavelength of 1564.4 nm is shown in Fig. 7b. Comparing the fields with the ones for the two bilayers of TiO$_2$/Au, the amplitude of the field for the TM wave is greater, but for the TE wave is smaller.

**Fig. 8:**
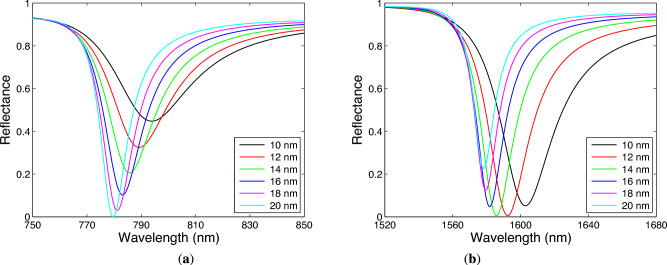
Theoretical spectral reflectances for TM (**a**) and TE (**b**) waves for the thickness of Au in a range of 10–20 nm when two bilayers of TiO$_2$/Au are considered. The angle of incidence $\theta =41.9^\circ$ and the analyte RI $n=1$.

**Fig. 9:**
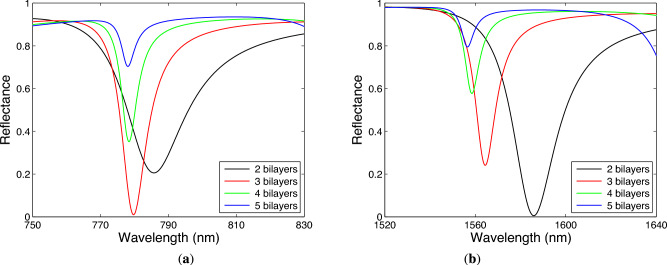
Theoretical spectral reflectances for TM (**a**) and TE (**b**) waves for the different number of bilayers of TiO$_2$/Au. The Au thickness $t_\textrm{m}=14$ nm, the angle of incidence $\theta =41.9^\circ$ and the analyte RI $n=1$.

The width and depth of the dips depend also on both the metal thickness $t_\textrm{m}$ and the number of bilayers of TiO$_2$/Au. Considering the analyte RI $n=1$ and the angle of incidence $\theta =41.9^\circ$, in Fig. 8a,b, the short-wavelength and long-wavelength dips corresponding to the Bloch-like SW resonances are shown for the thickness of Au in a range of 10–20 nm when the two bilayers of TiO$_2$/Au are taken into account. For the BS-like SW resonance of the TM wave shown in Fig. 8a, the dip width decreases and the dip depth increases as the thickness of Au increases. For the Bloch-like SW resonance of the TE wave shown in Fig. 8b, the dip width also decreases, but its depth increases up to 14 nm thickness of Au and further decreases with the Au thickness. It should be stressed that contrary to the SPR where the depth decreases and the width increases with the Au thickness, the resonance curves depicted in Fig. 8a exhibit opposite behavior. In Fig. 9a,b, the short-wavelength and long-wavelength dips, corresponding to the Bloch-like SW resonances of the TM and TE waves respectively, are shown for the different number of bilayers of TiO$_2$/Au, and the dip width decreases as the number of bilayers of TiO$_2$/Au increases. However, the dip depth also decreases for the Bloch-like SW resonance of the TE wave, but for the similar case of the TM wave, the dip depth increases up to three bilayers of TiO$_2$/Au and then it decreases with the number of bilayers.

Comparing the fields of the Bloch-like SWs in both TM and TE polarizations, as shown in Figs. 6 and 7, the penetration depth of the wave in the analyte is much longer for the TE wave than for the TM wave so that the field concentration in the analyte is higher thus justifying higher sensitivity to changes in the analyte RI. When comparing the fields with those for SPR- and all-dielectric 1DPhC-based sensors^26^, the penetration depth is the longest and the wave amplitude of the optical field at the analyte interface is the smallest for the SPR sensors, justifying the highest sensitivity and widest resonance dips. In all-dielectric 1DPhCs, the optical field at the analyte interface is more amplified, justifying the narrower resonance dips. In the metal-dielectric 1DPhCs, the extremely high RI contrast between the layers leads to superior field confinement and penetration in the analyte, and thus to the high sensitivity and FOM. Consequently, the metal-dielectric engineering of 1DPhCs can provide optical gas sensors with supreme parameters.

**Fig. 10:**
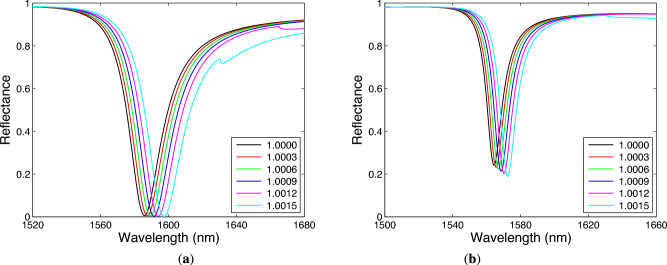
Theoretical reflectance for TE wave showing Bloch-like SW dip shift with analyte RI when two (**a**) and three (**b**) bilayers of TiO$_2$/Au are considered. The angle of incidence $\theta =41.9^\circ$.

To demonstrate the sensing properties of the metal-dielectric 1DPhC for the above chosen parameters, the computed spectral reflectance for the TE wave is analyzed for gaseous analytes with RI in a range of 1.000–1.0015 when two or three bilayers of TiO$_2$/Au are considered. The corresponding spectral reflectances are shown in Fig. 10a,b. It results from Fig. 10a that in the case of two bilayers of TiO$_2$/Au the dip shifts to longer wavelengths with no effect on its depth. In the case of three bilayers of TiO$_2$/Au, the dip depth is smaller but increases with the RI of analyte, as illustrates Fig. 10b, but the shift is smaller than that for the two-bilayer case.

**Fig. 11:**
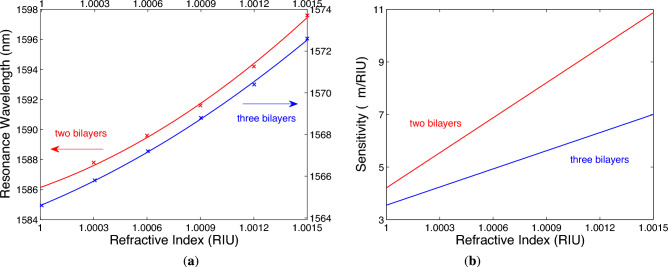
Resonance wavelength as a function of the analyte RI for different numbers of bilayers of TiO$_2$/Au with solid lines as fits (**a**). The corresponding sensitivity functions (**b**). The angle of incidence $\theta =41.9^\circ$, Au thickness $t_\textrm{m}=14$ nm, TiO$_2$ thickness $t_\textrm{d}=380$ nm and the termination layer thickness $t_\textrm{d}{'}=180$ nm.

The resonance wavelength $\lambda _\textrm{r}$ shown as a function of the RI *n* in Fig. 11a, is depicted together with fitting function (a second-order polynomial as the best fit) for both two and three bilayers of TiO$_2$/Au. Such a non-linear response means that the sensitivity to the refractive index $S_n$, defined as

11 $$\begin{aligned} S_n=\frac{\delta \lambda _\textrm{r}}{\delta n}, \end{aligned}$$

where $\delta \lambda _\textrm{r}$ is the change in the position of the dip with respect to the RI change $\delta n$ of the analyte, is linearly RI-dependent, as shown Fig. 11b. The sensitivities $S_n$ for two and three bilayers of TiO$_2$/Au are in a range of 4210–10,880 nm/RIU and 3550–7000 nm/RIU, respectively. Thus, they are higher as compared to a simple BSW-based sensor (up to 1456 nm/RIU^21^) or SPR-based gas sensors using metal-insulator-metal (MIM) structures^65–67^. It is interesting to note that from practical point of view, it is better to work in a sensing regime with a linear dependence of the resonance wavelength on the RI, which is attained at a greater angle of incidence. However, this regime is accompanied by the sensitivity decrease.

The sensor performance is also evaluated in terms of LOD and FOM. The LOD is defined as the smallest RI that can be reliably detected, and can be expressed by means of the smallest spectral shift $\delta \lambda _\textrm{min}$ that can be accurately measured and by the sensitivity $S_n$ as^68^

12 $$\begin{aligned} \textrm{LOD}=\frac{\delta \lambda _\textrm{min}}{S_n}. \end{aligned}$$

Based on the spectral resolution $\delta \lambda _\textrm{min}$ = 0.1 nm of a spectrometer, the LOD reaches for two and three bilayers of TiO$_2$/Au 9.3$\times$10$^{-6}$ RIU and 1.4 $\times$ 10$^{-5}$ RIU, respectively.

The FOM is defined as the sensitivity $S_n$ divided by the full-width half maximum (FWHM) of the resonance dip. The depth of the dip *D*, which is associated with the minimum reflectance, is a crucial factor in determining the FOM using the expanded definition^24^:

13 $$\begin{aligned} \textrm{FOM}=D\frac{S_n}{\textrm{FWHM}}. \end{aligned}$$

The FWHM of the resonance dips shown in Fig. 10a for the two bilayers of TiO$_2$/Au is approximately 23 nm and FOM is in a range of 183–474 RIU$^{-1}$. On the other hand, the FWHM of the resonance dips shown in Fig. 10b for the three bilayers of TiO$_2$/Au is approximately 12 nm, but even if the FOM is reduced by the depth *D* in a range of 0.8–0.82, it reaches values approximately from 240 to 485 that are slightly higher compared to those for the two-bilayer case.

**Fig. 12:**
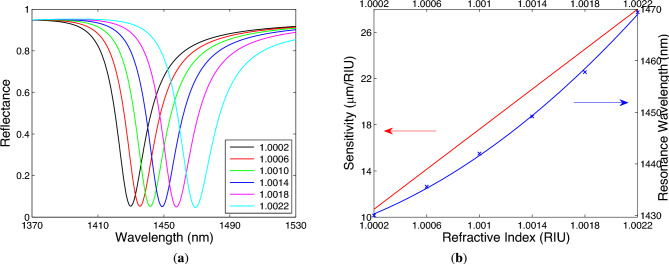
Theoretical reflectance for TM wave showing Bloch-like SW dip shift with analyte RI when two bilayers of TiO$_2$/Au are considered (**a**). Resonance wavelength as a function of the analyte RI and the corresponding sensitivity function (**b**). The angle of incidence $\theta =41.9^\circ$, Au thickness $t_\textrm{m}=14$ nm, TiO$_2$ thickness $t_\textrm{d}=400$ nm and the termination layer thickness $t_\textrm{d}{'}=340$ nm.

Because the chosen angle of incidence for the sensor is near critical angle for the generation of Bloch-like SW, the sensor can be operated in a limited range of RIs. To extend the measurement range at the same angle of incidence $\theta =41.9^\circ$, to sense for example nitrogen, whose RI changes in a range of 1.0002–1.0022^69^, parameters of the metal-dielectric 1DPhC need to be changed. As an example, analyzing the metal-dielectric 1DPhC with Au thickness $t_\textrm{m}=14$ nm, TiO$_2$ thickness $t_\textrm{d}=400$ nm and the termination layer thickness $t_\textrm{d}{'}=340$ nm, Fig. 12a shows the theoretical spectral reflectances for TM wave when two bilayers of TiO$_2$/Au are considered. In Fig. 12b, the resonance wavelength as a function of the RI is shown together with fitting function (a second-order polynomial), and from the same figure, in which the sensitivity $S_n$ is linearly RI-dependent, it results that it varies in a range of 10,680–28,000 nm/RIU. The corresponding LOD is as low as 3.6 $\times$ 10$^{-6}$ RIU. Taking into account the FWHM of the resonance dip with a value of 23 nm, the FOM varies in a range of 434–1217 RIU$^{-1}$.

Finally, we compare the performance of recent optical gas sensors from the point of view of the sensitivity and FOM, as presented in Table 1. This comparison highlights the distinctive advantage of the proposed optical sensors based on metal-dielectric 1DPhCs and supporting BLSWs. The performance is particularly notable when compared to sensors based on an CM in a 1DPhC^70^ or 2DPhC^71^, a PhC and plasmonic (PhC-P) hybrid topology^72^, a plasmonic structure with a semicircular ring resonator containing Ag NDs and an MIM waveguide (P-MIM-WG)^65^, a Fano resonance-based sensor comprising an MIM-WG^66^, an MIM WG with a perpendicularly coupled concentric triple ring resonator (CTRR)^73^, and a PhC micro-ring resonator (PhC-MRR)^74^. However, sensors outperforming the BLSW-based sensors are based on an CM^38^ or TPs in 1DPhCs with porous silicon (PSi)^47^ or gallium nitride (PGaN)^75^ layers and thus have some experimental limitations.

**Table 1:** Comparison of recent optical gas sensors.

Method	RI range	Sensitivity (nm/RIU)	FOM (RIU$^{-1}$)	Year
CM (1DPhC)	1.0005087–1.0005387	21,688	–	2019^70^
TP (PSi PhC)	1.00026–1.00046	190,000	360,000	2020^47^
TP (PGaN PhC)	1.00–1.01	28,753	66,000	2021^75^
BSW and GM	1.000–1.010	1456	91	2021^21^
PhC-P	1.000376–1.001132	1250	2,083	2022^72^
MIM-WG-CTRR	1.00–1.10	3639	91	2022^73^
CM PSi	1.0001–1.0005	52,300	402,300	2023^38^
P-MIM-WG	1.000–1.010	2900	40	2023^65^
MIM-WG	1.000–1.010	1450	242	2023^66^
MIM-WG	1.000–1.010	1450	242	2023^66^
PhC-MRR	1.00027300–1.00074873	6500	2,960	2024^74^
CM (2DPhC)	1.00037–1.00075	264	273	2025^71^
BLSW	1.0000–1.0015	10,900	474	This work
BLSW	1.0002–1.0022	28,000	1217	This work

## Conclusions

In this paper, a concept for sensing gaseous analytes based on the Bloch-like SW-based resonances supported by a metal-dielectric 1DPhC in the Kretschmann configuration is presented. For a metal-dielectric 1DPhC comprising different numbers of bilayers of TiO$_2$/Au with a termination layer of TiO$_2$, the reflectance responses have been analyzed theoretically and gas sensing ability based on the Bloch-like SW resonances in both TM and TE polarizations have been revealed. Using the wavelength interrogation and the RI changes in a range of 1–1.0015, the sensitivity, FOM, and LOD of 10,900 nm/RIU, 474 RIU$^{-1}$, and 9.3 $\times$ 10$^{-6}$ RIU, respectively, have been attained for the TE wave. The analysis extended to RI changes in a range of 1.0002–1.0022 employed a metal-dielectric 1DPhC with the modified the thicknesses of TiO$_2$ layers, and an ultra-high sensitivity up to 28,000 nm/RIU, FOM up to 1217 RIU$^{-1}$, and a very low LOD of 3.6 $\times$ 10$^{-6}$ RIU have been reached for the TM wave.

This sensing concept, based on the use of the multilayer metal-dielectric structures that are mechanically and chemically robust, is demonstrated in the field of ultra-high sensitivity gas sensors. The sensing concept is also offering the possibility of operation in aggressive environments and is advantageous because porous layers are not employed so that the response time of the sensor should be as short as possible. Moreover, the design of sensors employing a metal-dielectric 1DPhC supporting Bloch-like SWs can be optimized to allow for a simple sensing of a wide range of gaseous analytes. Finally, two-dimensional materials such as black phosphorus^76^ are supposed to be included in the sensor with the effect of increasing the sensitivity and extending applicability of optical sensors to special gaseous analytes.

## Data Availability

Data underlying the results presented in this paper are openly available from a Zenodo data repository at https://doi.org/10.5281/zenodo.17976043.
